# LncRNA *GAS8-AS1* is downregulated, correlates with early-stage disease and lymph node metastasis, and its knockdown promotes proliferation, migration, and invasion in differentiated thyroid cancer

**DOI:** 10.3389/fendo.2026.1839154

**Published:** 2026-06-08

**Authors:** Avaniyapuram Kannan Murugan, Hindi Al-Hindi, Ali S. Alzahrani

**Affiliations:** 1 Division of Molecular Endocrinology, Department of Molecular Oncology, King Faisal Specialist Hospital and Research Centre, Riyadh, Saudi Arabia; 2 Department of Pathology and Laboratory Medicine, King Faisal Specialist Hospital and Research Centre, Riyadh, Saudi Arabia; 3 Department of Medicine, King Faisal Specialist Hospital and Research Centre, Riyadh, Saudi Arabia

**Keywords:** DTC, *GAS8-AS1*, gene expression, human cancer, invasion, lncRNA, migration, proliferation

## Abstract

**Background:**

LncRNAs emerge as critical regulators of gene expression and epigenetic modulation in human cancer. However, the biological and clinical significance of the lncRNA *GAS8-AS1* in differentiated thyroid cancers (DTCs) remains poorly understood.

**Methods:**

*GAS8-AS1* expression in normal tissues was evaluated using LncExpDB. Quantitative RT-PCR was performed on 21 DTCs with matched adjacent normal tissues. RNA-seq data from TCGA (507 DTCs) were analyzed to assess *GAS8-AS1* expression and clinicopathological associations. Independent validation was conducted using ENCORI (510 thyroid cancer, 58 normal) and TNMplot datasets. Expression profiles of *GAS8-AS1*-associated genes were analyzed in TCGA and were corroborated with ENCORI dataset. Co-expression analyses were performed to identify regulatory relationships. Functional characterization was conducted using siRNA-mediated knockdown of *GAS8-AS1* in HEK293T and BCPAP cells, and overexpression studies in papillary thyroid cancer cell lines (K1 and BCPAP). Cell proliferation, migration, and invasion assays were performed. Pathway enrichment analyses were used to identify *GAS8-AS1*-mediated biological processes.

**Results:**

*GAS8-AS1* expression was significantly downregulated in DTCs compared with matched normal tissues (*p* < 0.0001). TCGA analysis confirmed lower *GAS8-AS1* expression, which was markedly associated with early-stage disease (*p* = 0.03) and lymph node metastasis (*p* = 0.03). *GAS8-AS1* downregulation was remarkably consistent in thyroid cancer (ENCORI, *p* = 0.004). A dramatic downregulation of *GAS8-AS1* was also observed across pan-cancer, including thyroid cancer (TNMplot, *p* = 2.01 × 10^-128^). Expression analysis of *GAS8-AS1*-associated genes revealed frequent deregulation in DTCs (24%), including downregulation of *ATF2, ATG5, ATG7*, and *BECN1*, and upregulation of *NEAT1* and *UCA1*. Co-expression analysis revealed that *GAS8-AS1* and *ATG5* expression levels were positively correlated with *ATF2*, whereas *NEAT1* showed a negative association, suggesting *ATF2*-dependent transcriptional regulation of *GAS8-AS1*, *ATG5*, and *NEAT1*. Functional characterizations demonstrated that *GAS8-AS1* knockdown significantly increased proliferation, migration, and invasion, whereas *GAS8-AS1* overexpression markedly suppressed tumor cell proliferation. Pathway enrichment analyses implicated *GAS8-AS1*-related genes in autophagy, apoptosis, proliferation, invasion, and metastasis.

**Conclusion:**

These findings demonstrate that *GAS8-AS1* may function as a tumor suppressor in DTCs, with its downregulation associated with disease progression and metastasis. The consistent loss of *GAS8-AS1* expression and its functional impact on tumor progression suggest that it may serve as a valuable diagnostic and prognostic biomarker in DTCs.

## Introduction

Thyroid cancer is the most prevalent malignancy of the endocrine system, and its incidence has consistently increased over the last forty years (1). It predominantly affects young women, representing the fifth most common cancer among females worldwide and the second most common cancer among women in Saudi Arabia (2, 3). According to the World Health Organization (WHO), thyroid cancer is classified into multiple subtypes, such as papillary (PTC), follicular (FTC), oncocytic, poorly differentiated (PDTC), and anaplastic (ATC) thyroid cancer, and all of them arise from thyroid follicular epithelial cells. In contrast, medullary thyroid carcinoma (MTC) originates from parafollicular C cells and accounts for fewer than 5% of all thyroid cancers. Papillary and follicular thyroid carcinomas, together with their respective variants, are collectively classified as differentiated thyroid cancer (DTC) and account for approximately 80–85% of all thyroid malignancies (4). Current therapies for thyroid cancer primarily include surgical resection of the tumor, radioactive iodine (RAI) therapy for iodine-avid tumors, and thyroid-stimulating hormone (TSH) suppression using levothyroxine. Targeted therapies, such as tyrosine kinase inhibitors (TKIs), are employed for advanced or RAI-refractory cases. External beam radiotherapy and chemotherapy are occasionally used for anaplastic or aggressive malignancies (5). Although most DTCs display low metastatic potential and have an excellent prognosis, with survival rates of approximately 90–95%, a subset demonstrates aggressive, highly metastatic behavior and resistance to current therapies (6). Despite substantial progress in elucidating the molecular mechanisms of thyroid cancer, significant knowledge gaps remain, underscoring the need for further research to fully understand its pathogenesis.

The mitogen-activated protein kinase (MAPK) and phosphatidylinositol 3-kinase/AKT/mTOR (PI3K/AKT/mTOR) pathways are the key signaling cascades involved in the pathogenesis of DTC (4). This process involves a spectrum of genetic alterations, including point mutations and gene rearrangements. For example, the BRAF (V600E) mutation and RET/PTC fusions in PTC, as well as *NRAS* mutations and *PAX8/PPAR-γ* rearrangements in FTC. In addition, mutations in the *TERT* promoter, *PIK3CA*, *PTEN*, *EGFR*, *CHEK2*, *EIF1AX*, and *PPM1D* have been identified, along with less frequent alterations in other genes (7–13). Furthermore, The Cancer Genome Atlas (TCGA) study of thyroid cancer has identified more than 95% of the candidate driver genes implicated in DTCs (12). Subsequent studies have identified additional genetic alterations associated with PDTCs and ATCs (14–16). Moreover, several genes have been found to be differentially expressed in thyroid cancers, with notable underexpression of *TP53, PTEN, CDKN1B*, and *TUSC2* frequently observed across various subtypes, including PTCs, FTCs, and ATCs (17–19). This downregulation has been shown to impact the thyroid-stimulating hormone receptor (TSHR) and the sodium/iodide symporter (NIS), leading to a loss of differentiated cellular characteristics (20). Furthermore, promoter hypermethylation, histone modifications of various regulatory protein-coding genes, and changes in expression/genetic alterations of noncoding RNAs (ncRNAs) represent key epigenetic mechanisms that suppress the expression of these genes. This disruption impairs essential cellular processes, including cell cycle regulation, apoptosis, DNA repair, and cell adhesion, thereby facilitating uncontrolled proliferation and tumor invasion (21).

Although over 90% of the mammalian genome is transcribed, less than 2% of these transcripts are translated into proteins. The majority of the remaining RNA molecules lack protein-coding potential and remain untranslated, collectively referred to as ncRNAs (22). The ncRNAs regulate gene expression at multiple levels, including splicing, transcription, and post-transcriptional processes. They also play a pivotal role in epigenetic regulation through mechanisms such as heterochromatin formation, histone modification, targeted DNA methylation, and gene silencing (23). Based on their size, ncRNAs are generally classified into two main groups: short noncoding RNAs (sncRNAs), which are under 200 nucleotides, and long noncoding RNAs (lncRNAs), which exceed 200 nucleotides in length. Although lncRNAs could range from 200 nucleotides to 100 kb, they are typically shorter than mRNAs due to having fewer exons. LncRNAs are crucial in epigenetically regulating genes involved in key cellular processes, including mesenchymal stem cell differentiation, cell cycle control, proliferation, autophagy, apoptosis, migration, and invasion (24). In cancer, lncRNAs function as tumor suppressors, oncogenes, or regulatory molecules across various human malignancies, including thyroid cancer (25).

**Figure 1:**
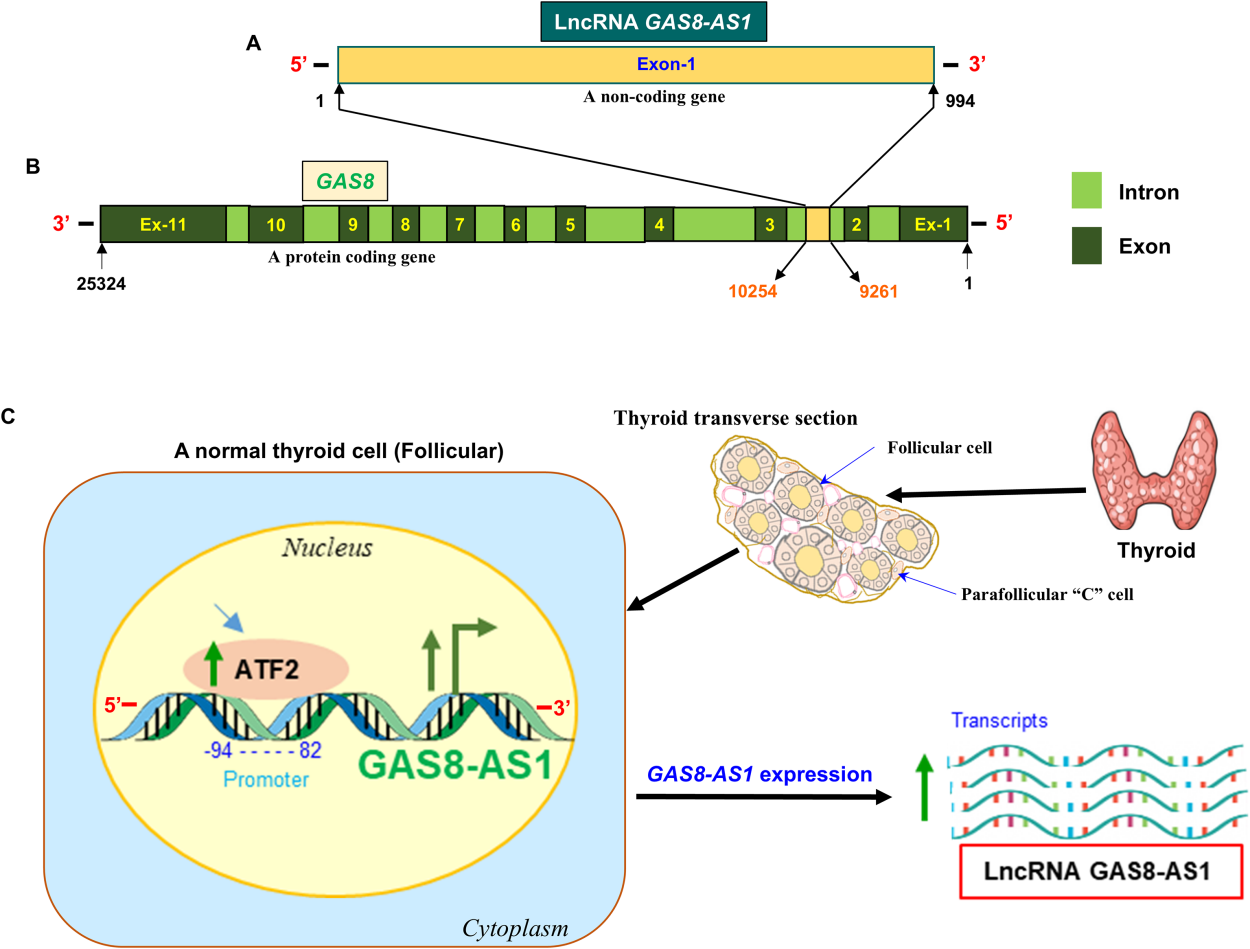
Schematic diagram of lncRNA *GAS8-AS1* and its transcription mechanism in thyroid cells. **(A)** Schematic diagram of the lncRNA *GAS8-AS1* gene displaying only one exon consisting of 994 nucleotides, and is shown in a sense (5’- 3’) orientation. **(B)** The illustration shows the schematic structure of the *GAS8* gene and is displayed in an antisense (3’- 5’) orientation, which exactly reflects the *GAS8-AS1* transcription from intron 2 (yellow color) of the *GAS8* gene. The exons and introns in the *GAS8* gene are indicated in dark and light green color, respectively. **(C)** Illustration shows the thyroid gland, transverse section of thyroid, and a thyroid cell indicating ATF2-mediated *GAS8-AS1* transcription.

Long noncoding RNA GAS8-antisense 1 (*GAS8-AS1*), located on chromosome 16, is transcribed from the intronic region (intron 2) of the coding gene *GAS8* (**Figure 1**). The *GAS8-AS1* has been reported to harbor frequent genetic alterations in Chinese PTC patients (26). Recently, we identified a dinucleotide genetic variant in *GAS8-AS1* that is significantly associated with the progression of DTCs, particularly in early-stage disease, as well as with lymph node and distant metastasis (27). Further, *GAS8-AS1* expression has been shown to be downregulated in pancreatic cancers, correlating significantly with lower five-year overall survival. Its expression was shown to clearly distinguish early-stage malignant cells from their normal counterparts (28). Conversely, overexpression of *GAS8-AS1* has been reported to inhibit hepatocellular carcinogenesis by epigenetically activating the tumor-suppressor gene *GAS8* (29). Similarly, *GAS8-AS1* overexpression suppresses colorectal cancer cell proliferation by downregulating *AFAP1-AS1* (30) and inhibits papillary thyroid cancer cell growth by enhancing ATG5-mediated autophagy (31).

Despite the high prevalence of DTCs in Saudi Arabia and the crucial epigenetic role of *GAS8-AS1* in various malignancies, its expression profile and clinical and functional significance in DTCs remain largely unexplored. In this study, we aimed to comprehensively elucidate the role of *GAS8-AS1* in DTCs. We analyzed its gene expression in DTC samples from Saudi Arabia and assessed RNA-seq data from TCGA to determine clinical correlations. Besides, *GAS8-AS1* expression was evaluated in thyroid cancer using ENCORI and across multiple cancers using TNMplot. We further investigated the dysregulation of *GAS8-AS1* and its associated upstream and downstream signaling genes in DTCs, along with their co-expression patterns. In addition, functional validation was performed *in vitro* using siRNA-mediated silencing in HEK293T and BCPAP cell lines. Furthermore, we overexpressed the mammalian expression vector (pcDNA3) carrying the lncRNA *GAS8-AS1* gene in PTC cells, including K1 and BCPAP, and assessed its role in cell proliferation and growth. Finally, we explored the major signaling pathways associated with *GAS8-AS1* and its related genes involved in vital cellular functions.

## Materials and methods

### Analysis of *GAS8-AS1* expression in normal tissues

Expression of the *GAS8-AS1* gene in normal tissues was assessed using RNA-seq data from the Expression Database of Human Long non-coding RNAs (LncExpDB) https://ngdc.cncb.ac.cn. The gene ID: HSALNG0113682 of *GAS8-AS1* was utilized to search the expression profiles of various normal organ tissues in the database, and the obtained *GAS8-AS1* expression values were plotted in descending order. The expression level was indicated in transcript per million (TPM).

### Differentiated thyroid cancer samples

In this study, we analyzed 21 fresh-frozen DTC tissue samples with their 21 matched adjacent normal tissues. Among the 21 malignant thyroid tumor samples, thirteen were classical PTC (CPTC), five were tall cell-PTC (TC-PTC), and three were follicular variant-PTC (FV-PTC). These samples were used to evaluate the expression of the *GAS8-AS1*. The tissue samples were collected from the Department of Pathology, King Faisal Specialist Hospital and Research Centre, Riyadh, Saudi Arabia. All the tissue samples were collected in 3 mL sample tubes with RNAlater solution (Ambion, USA), transported to the laboratory in cold storage, and the RNAlater solution was removed before the tissues were stored in −80 °C. Approval for this study was obtained from the institutional review board (IRB) (RAC# 2210017), and all experiments were performed following the prescribed guidelines of the IRB.

### Extraction of RNA from DTC tissues and complementary DNA synthesis

Frozen fresh tissue samples were thawed on ice and washed with ice-cold 1X PBS. A ~15–20 mg of the tissue sample was resected, added to the tubes containing Zirconium beads, and homogenized at 3000 rpm for 30 s. Total RNA was extracted using a commercially available RNeasy Mini Kit, Qiagen, USA (Cat. No. 74104), following the manufacturer’s instructions as previously described (26). With an intact 500 ng of total RNA, cDNA was synthesized using iScript cDNA Synthesis Kit, BIO-RAD, CA, USA (Cat. No. 1708890), and the obtained cDNA was stored at -20° C.

### Expression analysis of the *GAS8-AS1* using quantitative real-time PCR

The expression level of *GAS8-AS1* was analyzed in all DTC samples by qRT-PCR using cDNA synthesized as described above. The qRT-PCR reactions were performed in 96-well optical plates with 10 µl of cDNAs as a template and SYBR-Green master mix with *GAS8-AS1*-specific primers as mentioned previously (26). We used the *β-actin* gene as an endogenous control, and its primers were as described before (26). Thermal cycle conditions were as follows: an initial cycle of 50°C for 2 min and 95°C for 10 min, followed by 95°C for 15 s and 60°C for 1 min for 40 cycles using the 7500 Real-Time PCR system (Applied Biosystems, ThermoFisher Scientific, MA, USA). We performed all the reactions in triplicate, used mean Ct for analysis, and calculated the expression levels using the 2^− ΔΔ^*^CT^* method (31).

### Determination of *GAS8-AS1* expression in DTCs using TCGA data

The *GAS8-AS1* expression in the DTCs was analyzed by exploring the RNA-seq profiles in the TCGA data of Thyroid Carcinoma (TCGA, Firehose Legacy, 516 total samples) using cBioPortal (https://www.cbioportal.org) as previously described (32). We analyzed a total of 507 DTCs (399 PTCs, 107 FTCs, and 1 WDTC) for *GAS8-AS1* expression and correlated the expression level with disease stage and lymph node metastasis. We assessed the mRNA expression (z-scores) relative to all samples (log RNA-Seq V2 RSEM) with a z-score threshold of ±2.0.

### Analysis of *GAS8-AS1* expression in thyroid cancer from PANCAN datasets

We also determined the expression level of *GAS8-AS1* in 510 thyroid cancer and 58 normal samples from the PANCAN datasets using the ENCORI Pan-Cancer Analysis Platform for non-coding RNAs (https://starbase.sysu.edu.cn) as mentioned before (33).

### Evaluation of *GAS8-AS1* expression in pan-cancer

Differential gene expression of *GAS8-AS1* analysis in tumor and normal samples of pan-cancers was performed using TNMplot V2 (https://tnmplot.com/analysis/) as described previously (34). The TNMplot database consists of 56,938 unique samples from GEO, GTEx, TCGA, and TARGET repositories, comprising 15,648 normal and 40,442 tumors.

### Determination of the *GAS8-AS1*-associated gene expressions (*ATF2*, *ATG5*, *ATG7*, *BECN1* (Beclin 1), *NEAT1*, and *UCA1*) in 507 DTCs (TCGA) and 510 thyroid cancers (PANCAN)

Expression pattern of *ATF2, ATG5, ATG7, BECN1, NEAT1*, and *UCA1* was analyzed in the above-indicated RNA-seq data of 507 DTCs from TCGA using cBioPortal (https://www.cbioportal.org) as indicated before (32). Further, the aforementioned genes were also analyzed in 510 thyroid cancers and 58 normal samples using the ENCORI database (https://starbase.sysu.edu.cn) as described previously (33).

### Analysis of *GAS8-AS1, ATG5, ATG7, BECN1, NEAT1*, and *UCA1* gene expression within the ATF2 downregulated DTCs

The *GAS8-AS1, ATG5, ATG7, BECN1, NEAT1*, and *UCA1* gene expression was determined within the ATF2-downregulated DTCs from the TCGA data using cBioPortal (https://www.cbioportal.org) (32).

### Determination of co-expression pattern of *ATF2* vs *GAS8-AS1, ATG5*, and *NEAT1* in 507 DTCs

Co-expression patterns of *ATF2* vs *GAS8-AS1*, *ATF2* vs *ATG5*, and *ATF2* vs *NEAT1* were analyzed from the above-indicated 507 DTCs. Of a total of 507 DTCs, 6 cases were excluded as they were un-profiled/unavailable for co-expression data. These analyses were executed using TCGA datasets through cBioPortal (https://www.cbioportal.org) (32).

### Cell culture and reagents

We used HEK293T cells that were purchased from American Type Culture Collection (ATCC) (Cat. No. CRL-3216, Manassas, VA, USA). HEK293T cells were routinely cultured and maintained for experiments in Dulbecco’s Modified Eagle Medium (DMEM) (Cat. No. D6429-500ML, Sigma-Aldrich^®^, MO, USA) with 10% fetal bovine serum (FBS) (Cat. No. 16140-071, Gibco™, NY, USA), antibiotic, and antimycotic (Cat. No. 15240-062, Gibco™, NY, USA) per ATCC recommendations. The BCPAP cell line was cultured and maintained in the Roswell Park Memorial Institute 1640 (RPMI1640) medium (Cat. No. 11875-093, Gibco™, NY, USA) with 10% FBS (Cat. No. 16140-071) supplementing antibiotics and antimycotics (Cat. No. 15240-062). The BCPAP cells were authenticated as described previously (15).

### Transient knockdown of *GAS8-AS1* in HEK293T and BCPAP cells using siRNAs

Initially, we transiently transfected the HEK293T cells to knock down the *GAS8-AS1* expression. In brief, a day before the transfection, the HEK293T cells were plated in a 6-well plate, cultured without antibiotics and antimycotics. The next day, after the cells reach 70% confluence, we transfected 150 pmol of each of two different negative control siRNAs [Negative control siRNA-1 (NC-1), Cat. No. sc-37007; Negative control siRNA-2 (NC-2), Cat. No. sc-44230, Santa Cruz Biotechnologies, Inc., CA. USA] and two different *GAS8-AS1*-specific siRNAs (siRNA *GAS8-AS1*-1; siRNA *GAS8-AS1*-2) using Lipofectamine™ 2000 transfection reagent (Thermo Fisher Scientific, Inc., CA. USA) following the manufacturer’s instructions. The sense and antisense *GAS8-AS1*-specific siRNA sequences are exactly as mentioned previously (26). The same experiment was also subsequently and independently performed on BCPAP cells as was performed in HEK293T cells. Following transfection, both the HEK293T and BCPAP cells were incubated and maintained at 37 °C with 5% CO_2_ in standard conditions. These transiently transfected HEK293T and BCPAP cells were used in the downstream functional studies.

### RNA isolation from transfected human cells and cDNA synthesis

As indicated above, a part of each NC-1, NC-2, siRNA *GAS8-AS1*-1, and siRNA *GAS8-AS1–*2 transfected HEK293T/BCPAP cells was collected for total RNA isolation and cDNA synthesis as described before (35). In brief, 24 h after the transfection, HEK293T/BCPAP cells were collected and washed once with ice-cold 1X phosphate-buffered saline (PBS) pH 7.4. The cells were lysed, and total RNA was extracted from the transfected cells using TRIzol™ Reagent (Cat. No. 15596026, ThermoFisher Scientific, MA, USA). Each total RNA sample was treated with DNase (Cat. No. AM2222, Ambion™ DNase I (RNase-free), ThermoFisher Scientific, MA, USA) for the removal of genomic DNA. The quality and quantity of the extracted total RNA were analyzed by resolving in 1% standard native agarose gel and NanoDrop2000 (ThermoFisher Scientific, MA, USA), respectively. The cDNA was synthesized as described in the earlier paragraph of the “Materials and Methods” of this manuscript.

### Quantitative real-time PCR analysis of *GAS8-AS1* expression in siRNA-transfected HEK293T and BCPAP cells

The expression level of *GAS8-AS1* was analyzed in the cDNA prepared from negative control siRNA (NC-1; NC-2) and *GAS8-AS1*-specific siRNA (siRNA *GAS8-AS1*-1; siRNA *GAS8-AS1*-2) transfected HEK293T/BCPAP cells. The qRT-PCR reactions were carried out in 96-well optical plates using 10 µL of cDNA as the template, along with SYBR-Green master mix and *GAS8-AS1*-specific primers as described previously (26). We used *β-actin* as the endogenous control, employing primer sequences reported previously (26). The thermal cycling conditions included an initial incubation at 50 °C for 2 min and 95 °C for 10 min, followed by 40 cycles of 95 °C for 15 s and 60 °C for 1 min, performed on the Applied Biosystems 7500 Real-Time PCR System (Thermo Fisher Scientific, MA, USA). We performed all the reactions in triplicate, used mean Ct for analysis, and calculated the expression levels using the 2^− ΔΔCt^ method (36).

### Proliferation assay

Cell proliferation assay was performed as mentioned earlier (37). Briefly, as indicated above, both HEK293T/BCPAP cells were transiently transfected with negative control siRNAs (NC-1; NC-2) and *GAS8-AS1*-specific siRNAs (siRNA *GAS8-AS1*-1; siRNA *GAS8-AS1*-2). After 12 h, these transfected cells were washed once with 1X PBS, trypsinized, and seeded in triplicate with 4 × 10^3^ cells/well in 100 µl DMEM with 10% FBS in a 96-well plate (Cat. No. 3596, Costar, Corning Inc., NY) and cultured in standard conditions. The cell proliferation was measured using reagent WST-1 solution (Cat. No. 05015944001, Roche), 10 µL/well, and maintained at 37 °C with 5% CO2 for 4 h. All the samples were measured against a blank with absorption at 440 nm by a microplate reader (xMark Microplate Spectrophotometer, Bio-Rad).

### Wound healing assay

A wound healing assay was conducted as described previously (38). Briefly, HEK293T/BCPAP cells were transiently transfected with negative control siRNAs (NC-1; NC-2) and *GAS8-AS1*-specific siRNAs (siRNA *GAS8-AS1*-1; siRNA *GAS8-AS1*-2) as described earlier. At 24 h of transfection, cells were trypsinized, and an equal number of high concentrations of cells (2 × 10^5^) was plated in a 24-well cell culture plate. A scratch (0.6 mm) was made 6 hours after plating using a pipette tip. The wounded monolayer cell culture was washed thrice with 1 × PBS and maintained in standard conditions. We carefully watched the wound closure for 24 h from the initial scratch (0 h), measured, and photographed with x20 magnification under the microscope (OPTIKA XDS-3FL4, Italy). Wound healing percentage was calculated by measuring the scratch (gap) area at 0 h (initial scratch) and at 24 h time point using imageJ software from NIH, USA. The percentage of wound closure was determined using the formula: [(initial wound area-remaining wound area)/initial wound area] × 100. Results were expressed as percentage wound closure relative to time 0 h.

### Invasion assay

Cell invasion assay was performed as described previously (15, 16). In brief, HEK293T/BCPAP cells transiently transfected with negative control siRNAs (NC-1; NC-2) and *GAS8-AS1*-specific siRNAs (siRNA *GAS8-AS1-1*; *siRNA GAS8-AS1*-2) were serum starved for 8 h. At 24 h from the initial transfection, the cells were trypsinized, collected, and resuspended (5 × 10^4^ cells) in 500 µl of serum-free DMEM with 0.1% BSA and plated in Matrigel invasion chambers (culture inserts) consisting of 8-μm pore-size Matrigel matrix-coated polycarbonate filters (BD BioCoat™ Matrigel™ Invasion Chamber, BD Biosciences, Bedford, MA). The Matrigel chambers were inserted in a 24-well (BD Falcon) multi-well companion plate containing 750 µL of DMEM 0.5% serum. After 18 h of incubation in standard conditions, the invaded cells were fixed and stained as described before (15, 16).

### Construction of *GAS8-AS1* gene expression vector

Full-length open reading frame (ORF) of lncRNA *GAS8-AS1* gene (NR_122031.2) was PCR amplified from the complementary DNA (cDNA) of the HEK293T cells using a sense primer containing BamH1 (GAS8-AS1-BamH1-F: 5’ -ATCGCTGGATCCACCTGCAGTCCCAGCTAC - 3’) and an antisense primer containing EcoR1 (GAS8-AS1-EcoR1-R: 5’ - CACAGTGAATTCTCCTTGAACAATGGTAAA - 3’). The amplified DNA fragment was digested with restriction enzymes BamH1 and EcoR1, and the resulting fragment was cloned into a mammalian expression vector pcDNA3^®^ (Invitrogen Technologies, CA) using standard cloning strategies. The integrity of the plasmid containing GAS8-AS1 was verified by restriction enzyme digestion and sequencing. Plasmids were isolated for the transfection studies using the PureLink™ Quick Plasmid Miniprep Kit from Invitrogen, CA (Cat No: K2100–11).

### Papillary thyroid cancer cell lines and transfection

Papillary thyroid cancer cell lines, including K1 and BCPAP, were cultured in RPMI-1640 medium (Life Technologies, CA) with 10% fetal bovine serum (FBS) supplemented with penicillin G (100 u/mL), streptomycin (100 mg/mL), and maintained at 37 °C in a humidified incubator with 5% CO2. These cells were authenticated as described earlier (15). The K1 and BCPAP cells were transfected with each of pcDNA3-vector and pcDNA3-GAS8-AS1 using Lipofectamine 2000 as described in the previous paragraph in the “Materials and Methods”. At 12 h post-transfection, both K1 and BCPAP cells were trypsinized and reseeded into two separate cell culture plates: a 6-well plate, maintained under standard cell culture conditions for RNA isolation at 48 h post-transfection, and a 96-well plate for subsequent cell proliferation assays.

### RNA extraction, reverse transcription, and qRT-PCR

Total RNA extracted from the pcDNA3-vector and pcDNA3-GAS8-AS1 transfected BCPAP and K1 cells was extracted after 48 hrs later, cDNA synthesized, and *GAS8-AS1* expression was analyzed by qRT-PCR as described previously (26, 36). Detailed experimental procedures were provided in the earlier paragraph in “Materials and Methods”.

### Cell proliferation assay

BCPAP and K1 cells transfected with pcDNA3 vector or pcDNA3-GAS8-AS1 were trypsinized 12 h post-transfection, and 2 × 10³ cells were seeded into 96-well plates in triplicate, and cell proliferation assays were performed as described before (37). Detailed experimental procedures were mentioned in the earlier paragraph in “Materials and Methods”.

### Pathway enrichment analysis

Reactome version 95 (https://reactome.org/) analysis was carried out for all the pathways correlated with differentially expressed genes (DEGs) related to *GAS8-AS1* (*ATF2, ATG5, ATG7, BECN1, UCA1, NEAT1*, and *CTNNB1*) as described earlier (39). Total pathway reactions <5 were not considered and removed. In addition, we also analyzed pathway enrichment using the search tool for retrieval of interacting genes (STRING database, https://string-db.org) as described before (40). Protein–protein interaction (PPI) networks were constructed using active interaction sources such as text mining, experimental data, curated databases, and co-expression analyses. The analysis was restricted to Homo sapiens, and only interactions with a confidence score greater than 0.4 were included.

### Statistical analyses

Statistical analyses were performed using GraphPad Prism (version 8.0, GraphPad Software, USA). Continuous variables were analyzed using an unpaired Student’s *t*-test, while categorical variables were assessed using Fisher’s exact test or the Chi-square test, as appropriate; all tests were two-tailed. A *P*-value < 0.05 was considered statistically significant. Statistical significance between groups was indicated in the corresponding figures using asterisks, where n.s., *, **, and *** denote not significant, *P* ≤ 0.05, *P* ≤ 0.01, and *P* ≤ 0.001, respectively.

## Results

### The *GAS8-AS1* is ubiquitously expressed in normal tissues of various organs, including the thyroid gland

**Figure 2:**
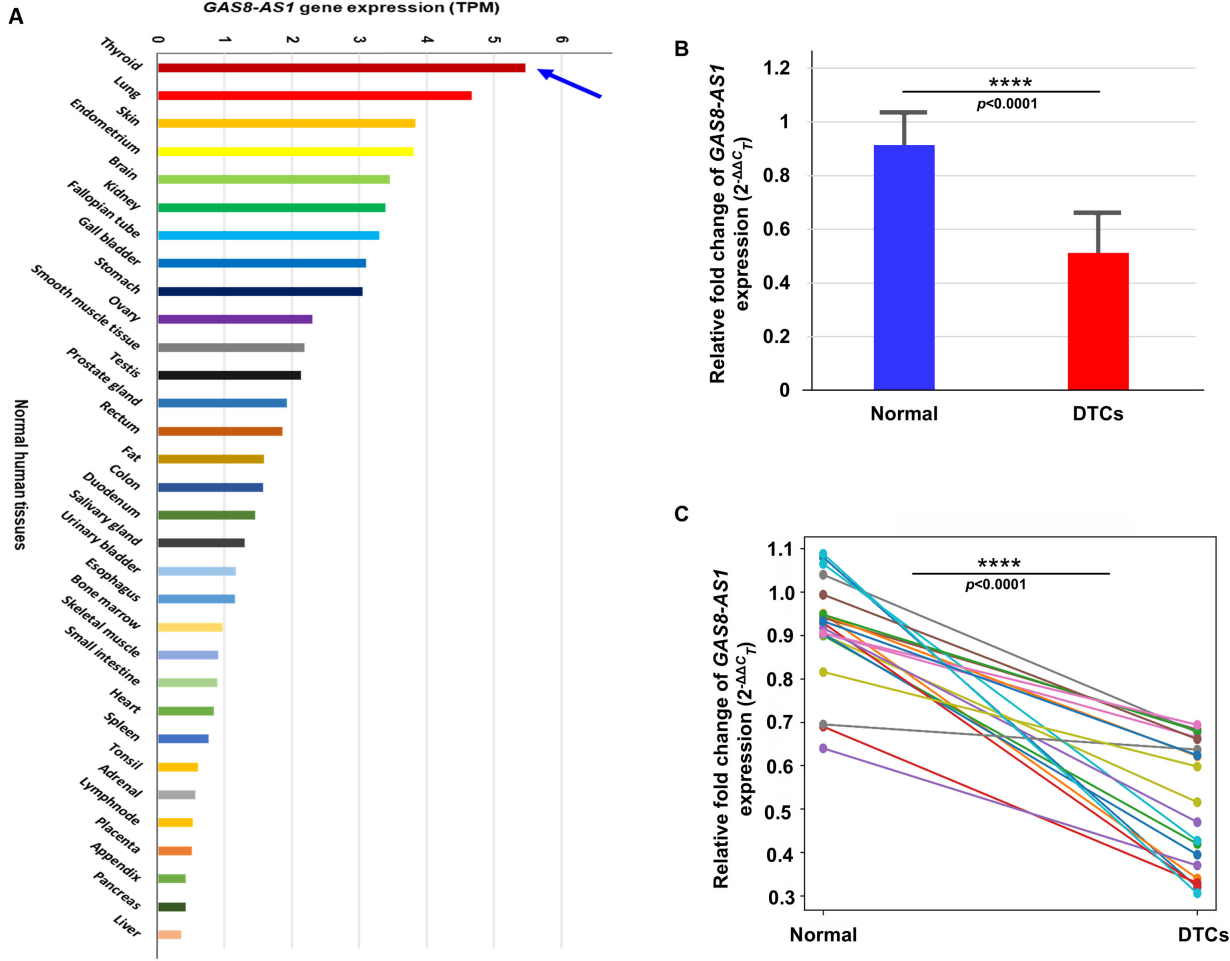
*GAS8-AS1* expression in normal and DTC tissues. **(A)** The bar diagram shows the expression level of *GAS8-AS1* gene in various normal human tissues, including the thyroid. The *GAS8-AS1* expression data for the normal tissues were collected from RNA-Seq data of the LncExpDB, analyzed, and the expression level of *GAS8-AS1* in various tissues is presented in descending order. Gene expression values are indicated in transcript per million (TPM). **(B)** The histogram shows the qRT-PCR analysis of *GAS8-AS1* expression in adjacent matched normal thyroid tissues and DTC tissues (n = 21), and the bar indicates the *GAS8-AS1* expression level in relative fold change. The *GAPDH* gene was utilized as an endogenous control. The 2^-δΔ^*^CT^* method was used to analyze the relative changes in *GAS8-AS1* expression from qRT-PCR experiments. Data were shown as means ± standard deviation (S.D.). The statistical difference between the normal tissues and DTCs was analyzed by Student’s *t-*test (paired), and the statistical significance is shown as an asterisk **** indicates *p* < 0.0001. **(C)** The illustration shows the paired-line and dot-plot, which indicates clearly the direction and magnitude of changes in *GAS8-AS1* expression for every single normal/DTC. The statistical significance marked as **** indicates *p* < 0.0001, as determined by the Wilcoxon signed-rank test. The presented data represent three independent experiments performed.

The tumor suppressive activity of *GAS8* has been shown to be exclusively dependent on *GAS8-AS1* in hepatocellular carcinomas (29). Besides, we found that *GAS8-AS1* bearing a dinucleotide variant causes a significant conformational change to the RNA secondary structure and is associated with early-stage DTCs and implicated in lymph node and distant metastasis (27). Hence, we hypothesized that *GAS8-AS1* might be downregulated in DTCs. Therefore, we were initially prompted to test whether the *GAS8-AS1* is expressed in normal human tissues. To determine the *GAS8-AS1* gene expression in various normal human tissues, we explored the Expression Database of Human Long non-coding RNAs (LncExpDB) using the gene ID of *GAS8-AS1*. As shown in **Figure 2A**, this comprehensive analysis found that the *GAS8-AS1* gene is ubiquitously expressed across 32 different kinds of normal human tissues, including tissue derived from the thyroid. The highest expression of *GAS8-AS1* was observed in normal thyroid tissues compared to the tissues derived from other organs, raising the possibility that *GAS8-AS1* has a potential function in the thyroid.

### The *GAS8-AS1* is significantly downregulated in DTCs

Expression levels of various lncRNAs have often been found to be deregulated in human cancer, including thyroid cancer (25). To determine whether *GAS8-AS1* expression is altered in DTCs, we performed qRT-PCR analysis on DTC samples (n = 21) and their matched adjacent normal tissues (n = 21). As shown in **Figure 2B**, *GAS8-AS1* was significantly underexpressed (1.8-fold) in DTC samples compared with their corresponding adjacent normal tissues (*p* < 0.0001). Moreover, as illustrated in **Figure 2C**, almost every paired line indicating *GAS8-AS1* expression level difference trends toward the same direction (normal-to-DTCs), which is a very strong effect (*p* < 0.0001), and the statistical test confirms that this is not due to random variation. Overall, as displayed in **Figure 2B, C**, our results demonstrate that *GAS8-AS1* is significantly downregulated in DTCs.

**Figure 3:**
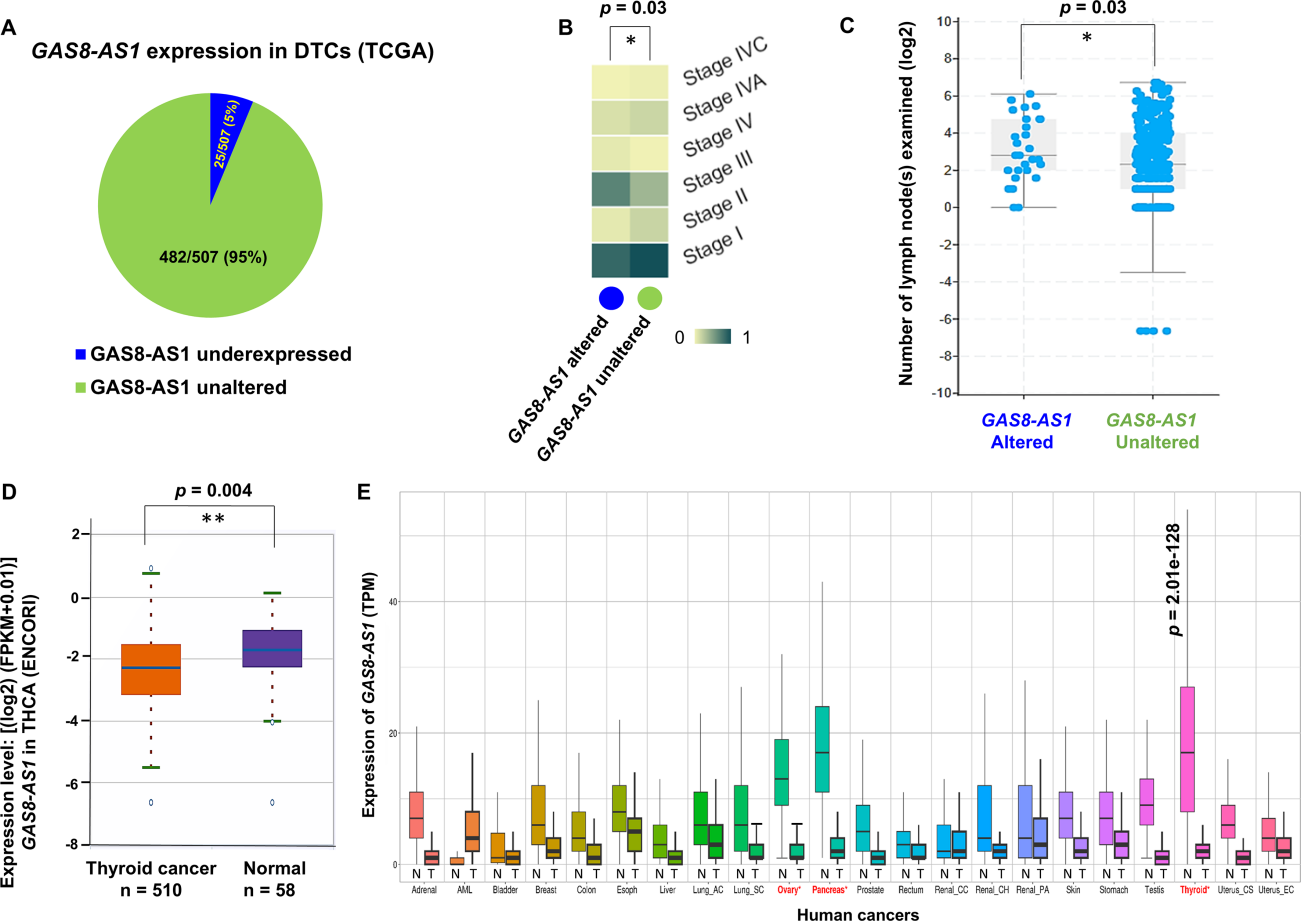
Analysis of *GAS8-AS1* expression in DTC tissues of TCGA datasets. **(A)** The pie chart shows *GAS8-AS1* expression in 507 DTCs. The number of underexpressed and unaltered cases among 507 DTCs is indicated in blue and light green color, respectively. The mRNA expression **(z-scores)** relative to all samples (log RNA-Seq V2 RSEM) with a z-score threshold of ±2.0. **(B)** Heatmap displays the association between tumor stages and *GAS8-AS1* expression. Tumor stages in DTCs were grouped into underexpressed and unaltered to see the association between them. DTC disease stage was determined as per the American Joint Committee on Cancer code. An asterisk (*) denotes a *p*-value of 0.03, as determined by the Chi-squared test. **(C)** The box plot shows the relationship between *GAS8-AS1* expression and lymph node metastasis. DTCs were grouped into underexpressed and unaltered to see the association between them. An asterisk (*) displays a *p*-value of 0.03, as determined by the Wilcoxon test. **(D)** Expression level of *GAS8-AS1* in thyroid cancer (n = 510) was compared with normal (n = 58) tissues in the data retrieved from the THCA dataset in ENCORI. Expression values were represented in reads per kilobase of transcript per million mapped reads (FPKM + 0.01). Data were shown as means ± standard deviation (S.D.). The asterisks (**) indicate *p* = 0.004, as determined by an unpaired *t*-test. **(E)** The pan-cancer box-and-Whisker plots displays the expression of the *GAS8-AS1* gene across normal and cancer tissues using RNA-Seq data of large datasets, including, TCGA, GEO and GTEx). In this box-and-whisker plot, center line shows median, box indicates interquartile range (25^th^ -75^th^ percentile), and 1.5 × the interquartile range **(IQR)** shows the range/Whiskers (used to identify outliers in data). In **Figure 3E**, *p* = 2.01e-128 indicates statistical significance, as determined by the Mann-Whitney test. *Cancer types highlighted in red show a statistically very significant deregulation of *GAS8-AS1* expression compared to the normal in those tumor types.

To corroborate this finding, we analyzed the RNAseq expression data of 507 DTCs from the TCGA datasets using cBioPortal. We found downregulation of *GAS8-AS1* expression levels in 4.9% (25/507) of DTCs (**Figure 3A**). Further, as shown in **Figure 3B, C**, the correlation of *GAS8-AS1* expression level with the clinicopathological features of DTCs revealed that the altered DTCs were markedly associated with early-stage disease (*p* = 0.03) and lymph node metastasis (*p* = 0.03). Consistent with these results, we also found the expression levels of *GAS8-AS1* were significantly lower (*p* = 0.004) in the thyroid cancer (n = 510) compared with those in normal samples (n = 58) in the Pan-cancer datasets, ENCORI (**Figure 3D**). These findings demonstrate that *GAS8-AS1* is downregulated in DTCs and thyroid cancer. Taken together, given the high prevalence of *GAS8-AS1* in DTCs, these results suggest that this may have a vital role not only in DTC pathogenesis but also in thyroid cancer.

### The *GAS8-AS1* expression is downregulated in various human cancers

In the present study, we initially identified a significant downregulation of *GAS8-AS1* in DTCs from the Saudi population. This finding was consistently replicated in independent datasets, including TCGA and ENCORI. Based on these observations, we further investigated whether *GAS8-AS1* is similarly downregulated across other human malignancies.

As illustrated in the box plot (**Figure 3E**) and summarized in **Table 1**, *GAS8-AS1* expression was significantly reduced in multiple cancer types, including adrenal carcinoma, acute myeloid leukemia (AML), bladder cancer, colon cancer, esophageal cancer, liver cancer, lung adenocarcinoma, lung squamous cell carcinoma, ovarian carcinoma, pancreatic cancer, prostate cancer, renal cell carcinoma, renal papillary carcinoma, renal chromophobe carcinoma, skin cancer, stomach cancer, testicular cancer, thyroid cancer, cervical squamous cell carcinoma, and endometrial carcinoma. Notably, the most dramatic downregulation of *GAS8-AS1* was observed in ovarian cancer (*p* = 1.77 × 10^-59^), pancreatic cancer (*p* = 1.03 × 10^-60^), and thyroid cancer (*p* = 2.01 × 10^-128^), highlighting a potentially broad tumor-suppressive role of *GAS8-AS1* across diverse malignancies.

**Table 1:** *GAS8-AS1* expression in human cancers.

S. no	Cancer type	Sample type	Analyzed sample size	*GAS8-AS1* expression Normal vs. Tumor *(p*-value) ^#^
1.	Adrenal	Normal	190	2.16e-26
		Tumor	79	
2.	AML	Normal	407	3.21e-65
		Tumor	151	
3.	Bladder	Normal	30	1.76e-01
		Tumor	411	
4.	Breast	Normal	403	3.19e-59
		Tumor	1097	
5.	Colon	Normal	274	2.20e-24
		Tumor	469	
6.	Esophagus	Normal	418	1.37e-13
		Tumor	161	
7.	Liver	Normal	225	1.93e-14
		Tumor	371	
8.	Lung-AC	Normal	486	3.10e-19
		Tumor	524	
9.	Lung-SC	Normal	476	1.43e-66
		Tumor	501	
10.	Ovary	Normal	133	1.77e-59
		Tumor	374	
11.	Pancreas	Normal	252	1.03e-60
		Tumor	177	
12.	Prostate	Normal	204	6.92e-32
		Tumor	498	
13.	Rectum	Normal	243	1.85e-09
		Tumor	166	
14.	Renal-CC	Normal	117	7.83e-01
		Tumor	535	
15.	Renal-CH	Normal	69	7.38e-06
		Tumor	65	
16.	Renal-PA	Normal	77	1.53e-01
		Tumor	289	
17.	Skin	Normal	474	8.96e-29
		Tumor	103	
18.	Stomach	Normal	294	5.22e-25
		Tumor	375	
19.	Testis	Normal	259	2.46e-60
		Tumor	156	
20.	Thyroid	Normal	504	2.01e-128
		Tumor	502	
21.	Uterus-CS	Normal	111	8.09e-19
		Tumor	56	
22.	Uterus-EC	Normal	146	1.18e-10
		Tumor	547	

^#^Mann-Whitney test; AML, acute myeloid leukemia; Lung-AC, lung adenocarcinoma; Lung-SC, lung small cell; Renal-CC, renal cell carcinomas; Renal-PA, renal papillary; Uterus-CS, uterus cervical squamous cell carcinoma; Uterus-EC, uterus endometrial carcinoma.

### *GAS8-AS1*-pathway genes’ expression is significantly deregulated in DTCs

**Figure 4:**
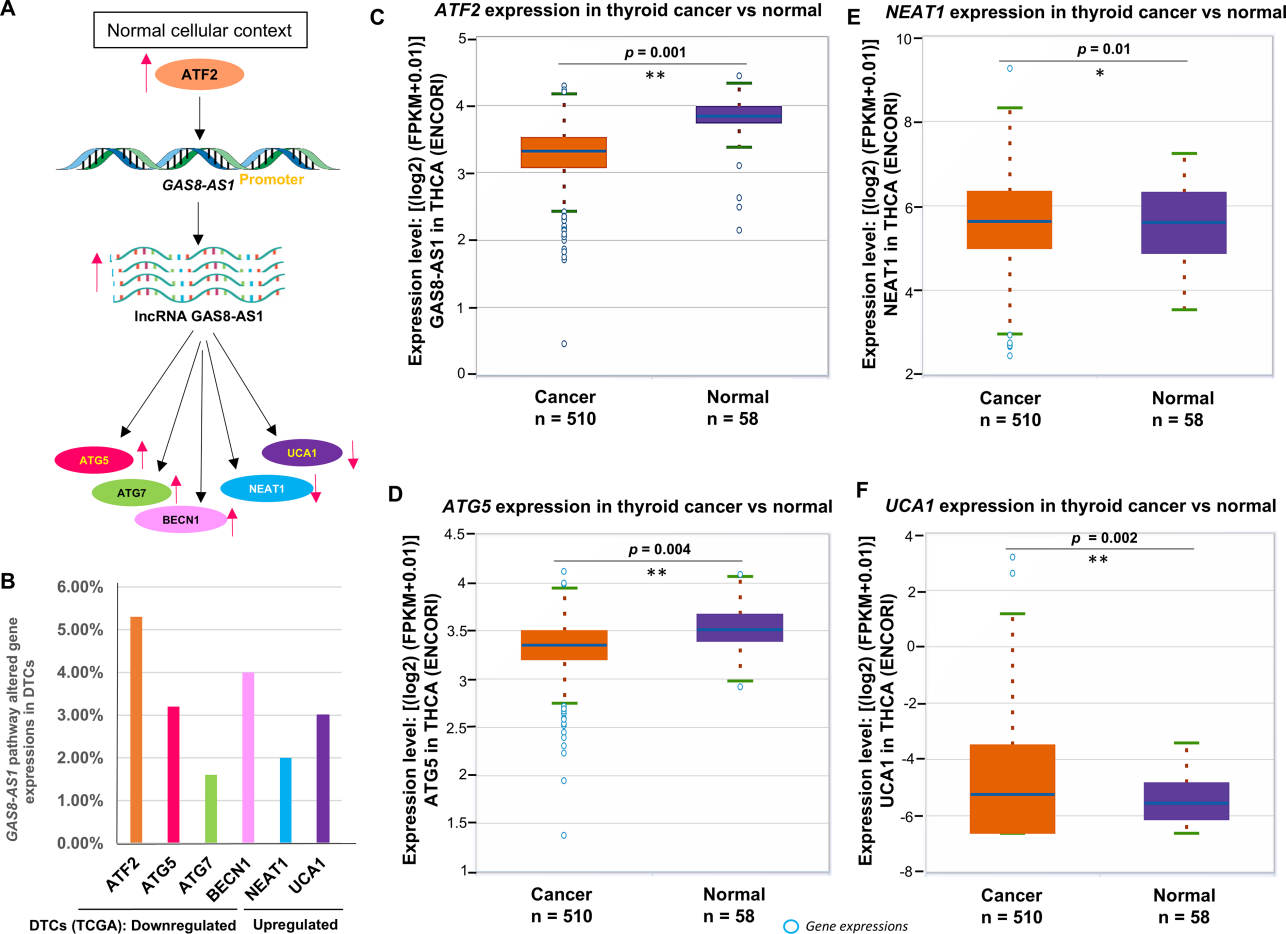
Deregulated expression pattern of *GAS8-AS1* pathway genes. **(A)** Schematic illustration indicates the *GAS8-AS1* pathway genes which include upstream *ATF2* and downstream *ATG5*, *ATG7, BECN1, NEAT1*, and *UCA1*. **(B)** Histogram shows the prevalence of deregulated expression (%) of *ATG5, ATG7, BECN1, NEAT1*, and *UCA1* genes in DTCs from TCGA (n = 507). **(C-F)** The box plot displays the expression pattern of *ATF2, ATG5, NEAT1*, and *UCA1*, respectively, in thyroid cancer (n = 510) compared with normal controls (n = 58) of THCA from ENCORI. The expression values are log2-transformed and indicated in FPKM + 0.01. Data were shown as means ± standard deviation (S.D.). Statistical significance is denoted as n.s., *, **, and ***, corresponding to “not significant”, *p* < 0.05, *p* < 0.01, and *p* < 0.001, respectively, as determined by an unpaired *t*-test.

The upstream *ATF2* has been shown to induce *GAS8-AS1* expression through *ATG5* and *ATG7* axes for promoting autophagy (41). Besides, *GAS8-AS1* was shown to activate the downstream *BECN1* to promote autophagy in ovarian cancer (42). The *GAS8-AS1* has also been found to downregulate the expression of *NEAT1* and *UCA1* to inhibit the proliferation, migration, and invasion of glioblastoma and osteosarcoma cells, respectively (43, 44). Therefore, we hypothesized that the expression of upstream (*ATF2*) and downstream effector molecules (*ATG5*, *ATG7*, *BECN1*, *NEAT1*, and *UCA1*) of *GAS8-AS1* may be deregulated (**Figure 4A**). To validate this, we initially examined the expression of *ATF2* in DTCs. We found that *ATF2* expression is downregulated in 5.3% (27/507) of DTCs. Subsequent analyses of downstream molecules of *GAS8-AS1* revealed that a significant downregulation of *ATG5* (3.2%, 16/507), *ATG7* (1.6% 8/507), *BECN1* (4%, 22/507), and upregulation of *NEAT1* (2%, 10/507) and *UCA1* (3%, 15/507) in DTCs (**Figure 4B**). Consistently, we also observed a remarkable downregulation of *ATF2* (*p* = 0.001) and *ATG5* (*p* = 0.004) and significant upregulation of *NEAT1* (*p* = 0.01) and *UCA1* (*p* = 0.002) in the thyroid cancer when compared to the normal samples in the pan-cancer datasets of ENCORI (**Figures 4C–F**) while *ATG7* and *BECN1* were differentially expressed (data not shown). Together, these results suggest that *GAS8-AS1* pathway genes are significantly deregulated in ~24% (123/507) of DTCs.

### *GAS8-AS1* promoter-binding protein, *ATF2* downregulation differentially modulates its downstream effector genes in DTCs

**Figure 5:**
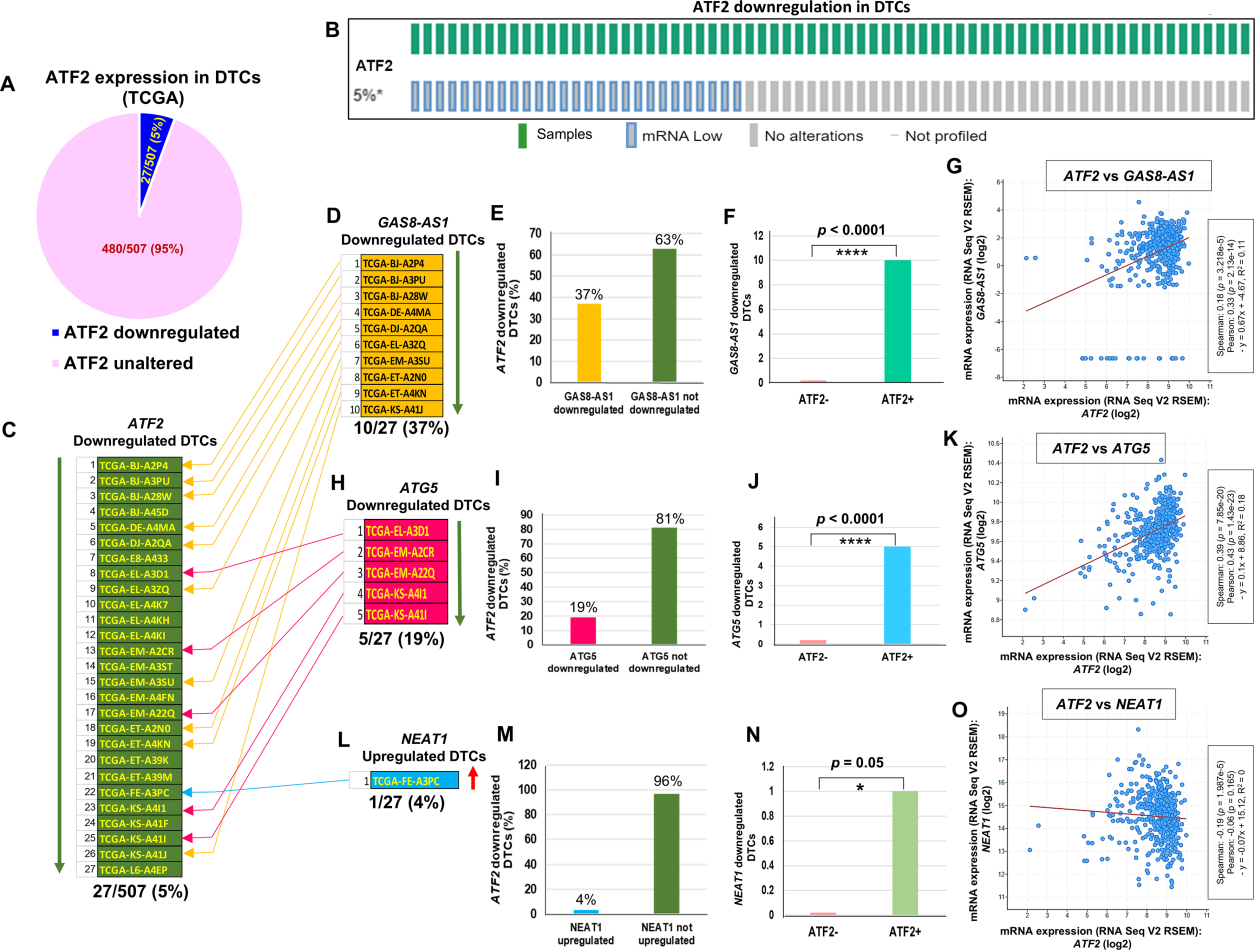
*ATF2*-dependent expression pattern of *GAS8-AS1, ATG5*, and *NEAT1* in DTCs. **(A)** Pie chart illustrates the *ATF2* expression in DTCs (n = 507). The percentage of *ATF2*-downregulated and *ATF2*-unaltered DTC cases is shown in blue and pink color, respectively. **(B)** Oncoprint indicates the percentage of *ATF2* downregulation in DTCs (5% (27/507). **(C)** The list in green color indicates *ATF2*-downregulated DTCs. All 27 *ATF2* downregulated DTCs are shown with sample numbers (TCGA). **(D)** List in yellow color displays *GAS8-AS1* downregulation within the 27 *ATF2*-downregulated DTCs. **(E)** Histogram indicates the percentage of *GAS8-AS1* downregulation (37%, 10/27) within the *ATF2* downregulated cases n = 27, (100%). **(F)** Downregulation was observed in 10 of 27 ATF2-positive cases (37%), whereas none of the 480 ATF2-negative cases showed downregulation. This association was highly significant (Fisher’s exact test, ****p < 0.0001). **(G)** Correlation between *ATF2* and *GAS8-AS1* in DTCs. **(H)** List in pink color shows ATG5-downregulated samples within the 27 *ATF2* underexpressed DTCs. **(I)** The histogram shows the percentage of *ATG* downregulation (19%, 5/27) within the *ATF2* downregulated cases. **(J)** Downregulation was observed in 5 of 27 ATF2-positive cases (19%), whereas none of the 480 ATF2-negative cases showed downregulation. This association was highly significant (Fisher’s exact test, ****p < 0.0001). **(K)** Correlation between *ATF2* and *ATG5* in DTCs. **(L)** List in blue color displays *NEAT1*-upregulated sample within the 27 *ATF2* underexpressed DTCs. **(M)** The bar diagram shows the percentage of *NEAT1* upregulation (4%, 1/27) within the *ATF2* downregulated cases. **(N)** Downregulation was observed in 1 of 27 *ATF2*-positive cases (3.7%), whereas none of the 480 ATF2-negative cases showed downregulation. This difference showed a trend toward significance (Fisher’s exact test, *p = 0.05). **(O)** Correlation between *ATF2* and *NEAT1* in DTCs. The *p*-value < 0.05 was considered statistically significant.

ATF2 was demonstrated to bind in the promoter region (-82-94) of *GAS8-AS1*, and its expression was shown to be critical for the transcription of *GAS8-AS1* (41). Therefore, we speculated that if *ATF2* is downregulated, the downstream molecules, including *GAS8-AS1*, *ATG5*, *ATG7*, *BECN1*, *NEAT1*, and *UCA1*, would also be deregulated in an *ATF2*-dependent manner. To examine this, we analyzed the expression of these molecules within the 27 *ATF2*-downregulated DTCs (**Figures 5A–C**). As illustrated in **Figures 5D–F**, our analysis revealed that the *GAS8-AS1* was significantly downregulated in 10/27, 37% (*p* < 0.0001) of *ATF2*-downregulated DTCs, and also found a positive correlation between *ATF2* and *GAS8-AS1* expression (Pearson: *p* = 2.13e-14) (**Figure 5G**). Further analysis showed that the *ATG5* was remarkably downregulated in 5/27, 19% (*p* < 0.0001) of *ATF2*-downregulated DTCs (**Figures 5H–J**), and found a significant positive correlation between *ATF2* and *ATG5* expression (*p* = 1.43e-23) (**Figure 5K**). As shown in **Figures 5L–N**, the *NEAT1* was upregulated in 1/27, 4% (*p* = 0.05) *ATF2*-downregulated DTCs, and there was a negative correlation between *ATF2* and *NEAT1* expression (*p* = 0.165) (**Figure 5O**). However, we did not observe any ATF2-dependent expression pattern for *ATG7*, *BECN1*, and *UCA1*. These results indicate that underexpression of *ATF2* could lead to the downregulation of *GAS8-AS1* and *ATG5*, while upregulating *NEAT1*. Taken together, these findings suggest that *ATF2* may be associated with the expression of *GAS8-AS1*, *ATG5*, and *NEAT1* in DTCs.

### The siRNA-mediated suppression of *GAS8-AS1* expression enhances the proliferation of both HEK293T and BCPAP cells

**Figure 6:**
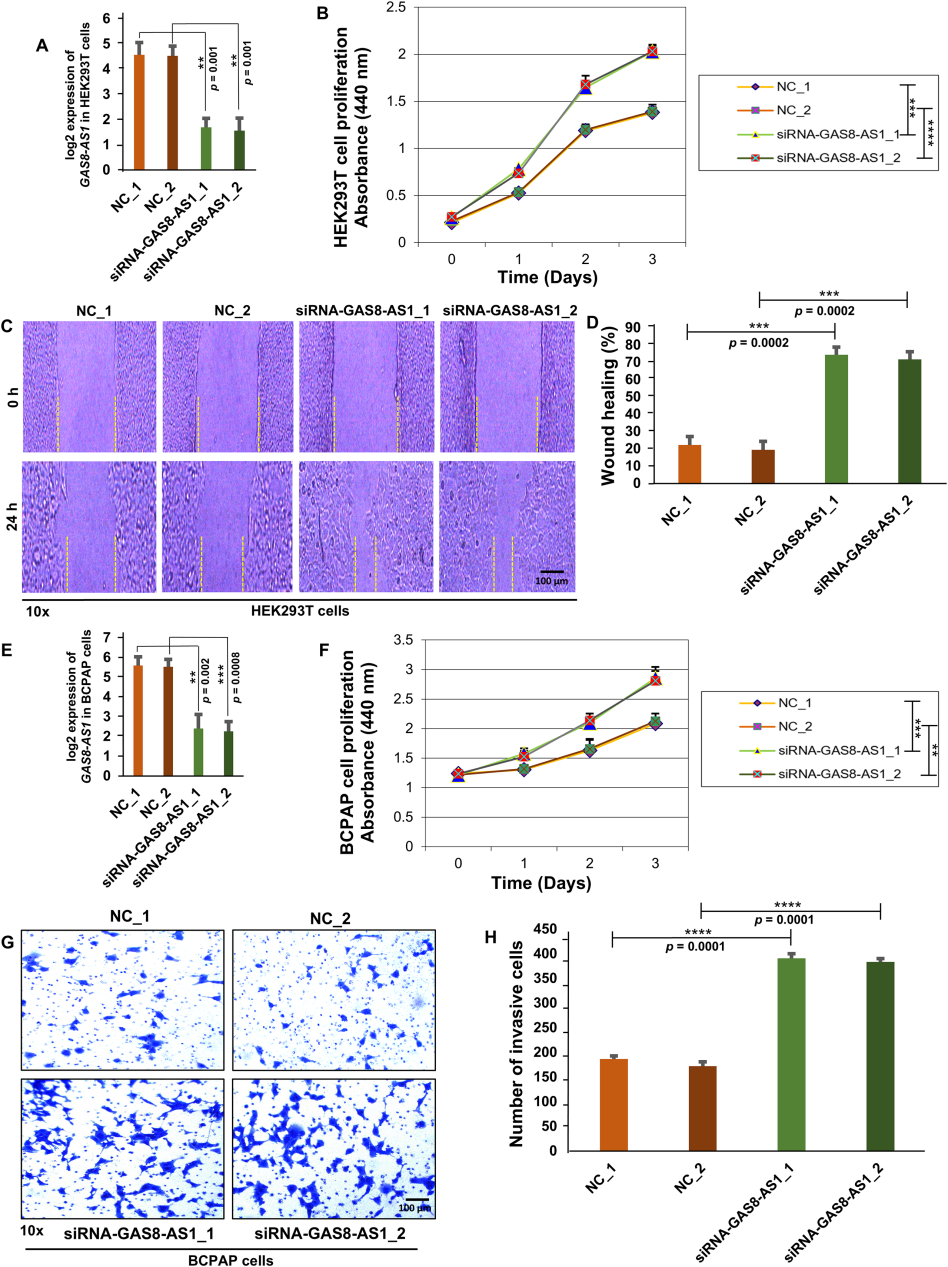
siRNA-mediated knockdown of *GAS8-AS1* promotes cell proliferation, migration, and invasion in HEK293T and BCPAP cells. **(A)** Histogram displays the *GAS8-AS1* expression level in HEK293T cells after transfection of scrambled siRNAs (NC_1 and NC_2) and *GAS8-AS1*-specific siRNAs (siRNA-*GAS8-AS*1_1 and siRNA-GAS8-AS1_2). **(B)** Cell proliferation of HEK293T cells transfected with the above-indicated negative control and *GAS8-AS1*-specific siRNAs. HEK293T cells were transiently transfected with the above-indicated negative controls (NC) and GAS8-AS1-specific siRNAs. The transfected HEK293T cells were plated in a 96-well plate in triplicate and maintained for the indicated days under standard conditions and their proliferation was evaluated. **(C)** Migration of HEK293T cells transiently transfected with indicated NC and *GAS8-AS1*-specific siRNAs in the wound healing assay. Representative wound closure images are shown for 0 and 24 h. **(D)** The histogram shows the percentage of wound healing. Cell migration in wound healing assay was calculated and expressed as the percentage of cell coverage of the initial cell-free zone in the scratch as detailed in “Materials and Methods”. **(E)** *GAS8-AS1* expression level in BCPAP cells after transfection of the above-indicated NCs and siRNAs. **(F)** Cell proliferation of BCPAP cells transfected with the indicated siRNAs. **(G)** An invasion assay of BCPAP cells with various siRNA transfections, exactly as indicated in **(A)** An invasion assay was conducted, followed by indicated transfections as explained in “Materials and methods”. The representative figure shows cells that invaded the Matrigel-matrix-coated PET (polyethylene terephthalate membrane) membrane. **(H)** The bar diagram shows the number of invasive cells. Values presented as means ± S.D. of three independent experiments. Statistical significance is displayed as n.s., *, **, ***, and **** indicate not significant, p < 0.05, p < 0.01, and *p* < 0.001, *p* < 0.0001, respectively as calculated by an unpaired *t-*test.

Overexpression of *GAS8-AS1* was shown to suppress various cell proliferation (29, 39). As we found the *GAS8-AS1* expression is downregulated in DTCs, we questioned whether suppression of endogenous *GAS8-AS1* could mimic its pathogenic function upon deregulation. To investigate the effect of *GAS8-AS1* downregulation, initially, we transiently transfected the HEK293T and BCPAP cells with two different *GAS8-AS1*-specific siRNAs (siRNA-*GAS8-AS1*_1 and siRNA-*GAS8-AS1*_2) and two different non-specific siRNAs (NC_1 and NC_2) as negative controls. HEK293T cells were used in addition to BCPAP cells primarily due to their high transfection efficiency and robust expression system, allowing a reliable preliminary assessment of *GAS8-AS1* function. Endogenous expression of *GAS8-AS1* was successfully suppressed in the two different *GAS8-AS1*-specific siRNA-transfected HEK293T and BCPAP cells but not in the cells transfected with negative controls, as shown in **Figures 6A, E**. To determine the level of proliferation of various siRNA-transfected, transiently silenced HEK293T and BCPAP cells, we performed proliferation assays. As shown in **Figures 6B, F**, *GAS8-AS1*-specific siRNA-transfected HEK293T and BCPAP cells exhibited dramatically increased cell proliferation compared to that of their parallel negative controls (NC_1 vs siRNA-GAS8-AS1_1 *** *p* < 0.001; NC_2 vs siRNA-GAS8-AS1 ****p < 0.0001). These results indicated that the *GAS8-AS1* could promote cell proliferation upon its downregulation, suggesting its pivotal role in DTC tumorigenesis.

### The siRNA-mediated inhibition of *GAS8-AS1* significantly increases migration and invasion of normal and papillary thyroid cancer cells, respectively

Metastasis is the main manifestation of migrating and invasive potentials of the malignant cells, and various lncRNAs have been implicated in accelerating invasion and metastasis (22, 25). The overexpression of *GAS8-AS1* was shown to reduce the motility of hepatic cancer cells (29). Therefore, we hypothesized that *GAS8-AS1* may play a vital role in the motility of DTCs. To further validate this, we transiently transfected two different *GAS8-AS1*-specific siRNAs with their paired negative controls in HEK293T cells, as described earlier, and performed a wound healing assay, the basic assay to measure the metastatic potential. As illustrated in **Figures 6C, D**, the *GAS8-AS1*-silenced HEK293T cells dramatically enhanced the wound healing and resulted in > 3-fold higher migration when compared to the cells bearing negative controls at a 24 h time period (NC_1 vs siRNA-GAS8-AS1_1 ***p < 0.0002; NC_2 vs siRNA-GAS8-AS1 ***p < 0.0002). As HEK293T cells transiently silenced with *GAS8-AS1*-specific siRNAs efficiently promoted wound healing, we speculated that they could also invade the Matrigel matrix-coated membrane that mimics cell metastasis. As shown in **Figure 6G**, BCPAP cells silenced with *GAS8-AS1*-specific siRNAs were 2-fold more invasive than the cells with negative controls, which were transfected with non-specific siRNAs (NC_1 vs siRNA-GAS8-AS1_1 ****p = 0.0001; NC_2 vs siRNA-GAS8-AS1 ****p = 0.0001). The number of invasive cells was 2-fold higher in the *GAS8-AS1*-specific siRNAs-transfected cells than in the cells transfected with non-specific siRNAs, negative control (**Figure 6H**). Taken together, these results indicate that *GAS8-AS1* promotes migration and invasion upon its downregulation, reflecting a prominent role in controlling the invasion and metastasis of DTCs.

### Overexpression of *GAS8-AS1* reduces the growth of papillary thyroid cancer cells, including K1 and BCPAP

**Figure 7:**
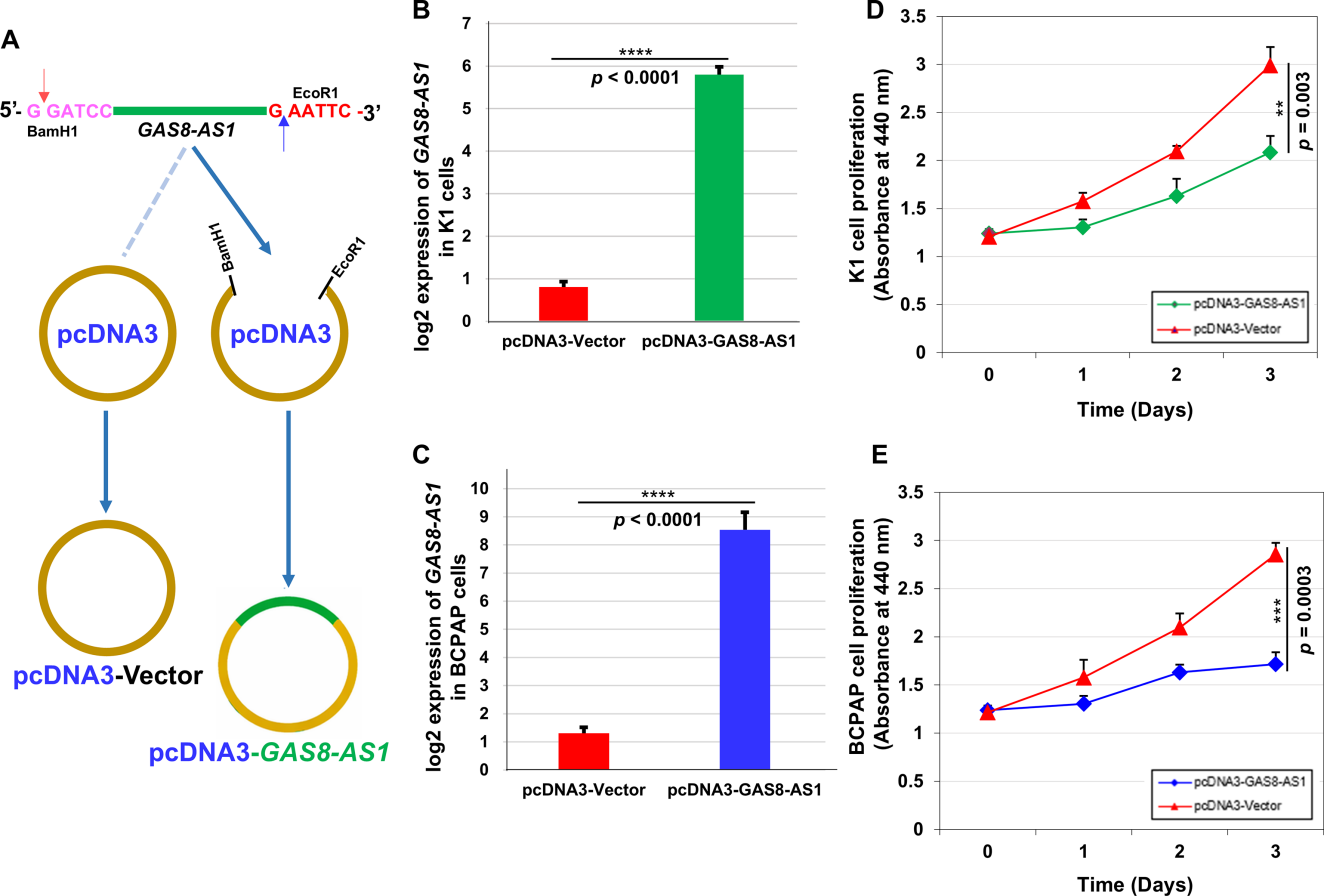
Overexpression of the *GAS8-AS1* gene in K1 and BCPAP thyroid cancer cells. **(A)** Schematic representation of the cloning of the *GAS8-AS1* gene into the mammalian expression vector pcDNA3. **(B, C)** Bar graphs showing the expression levels of *GAS8-AS1* in K1 and BCPAP cells following transient transfection with either empty vector (pcDNA3-vector) or GAS8-AS1-expressing vector (pcDNA3-GAS8-AS1). Total RNA was isolated from transfected cells, followed by cDNA synthesis and qRT-PCR analysis. Enhanced expression of *GAS8-AS1* was confirmed in pcDNA3-GAS8-AS1-transfected K1 and BCPAP cells. **(D, E)** Cell proliferation analysis of K1 and BCPAP cells transfected with pcDNA3-vector or pcDNA3-GAS8-AS1. Transfected cells were seeded in 96-well plates in triplicate, maintained under standard conditions for the indicated time points (days), and assessed for proliferation. Data are presented as mean ± S.D. from three independent experiments. Statistical significance is indicated as n.s. (not significant), *p* < 0.05 (*), *p* < 0.01 (**), *p* < 0.001 (***), and *p* < 0.0001 (****), determined by an unpaired *t*-test.

As we observed that *GAS8-AS1*-specific siRNA-transfected HEK293T and BCPAP cells exhibited dramatically increased cell proliferation compared to that of their parallel negative controls, we questioned whether overexpression of *GAS8-AS1* could decrease the proliferation of PTC cells. To validate this, as illustrated in **Figure 7A**, we cloned the *GAS8-AS1* gene into a mammalian expression vector (pcDNA3). Consequently, K1 and BCPAP cells were transiently transfected with either an empty vector (pcDNA3-vector) or a *GAS8-AS1*-expressing vector (pcDNA3-GAS8-AS1). Overexpression of *GAS8-AS1* likely could increase high level of lncRNA *GAS8-AS1* in transfected cells. Therefore, to evaluate its overexpression, total RNA was isolated from transfected cells, followed by cDNA synthesis and qRT-PCR analysis. We observed a significant increase (> 5-fold) of *GAS8-AS1* expression in K1 and BCPAP cells transfected with pcDNA3-*GAS8-AS1*, but vector-transfected control cells showed only an endogenous level of *GAS8-AS1* (**Figure 7B, C**). To determine the effect of vector and *GAS8-AS1* overexpression on K1 and BCPAP cells, we performed a cell proliferation assay. As demonstrated in **Figure 7D, E**, overexpression of *GAS8-AS1* significantly reduced the proliferation level of PTC cells, including K1 and BCPAP. Collectively, these findings suggest that *GAS8-AS1* may function as a tumor suppressor in DTCs, the most prevalent subtype of thyroid cancer.

### Identification of major signaling pathways associated with *GAS8-AS1*–related genes

**Figure 8:**
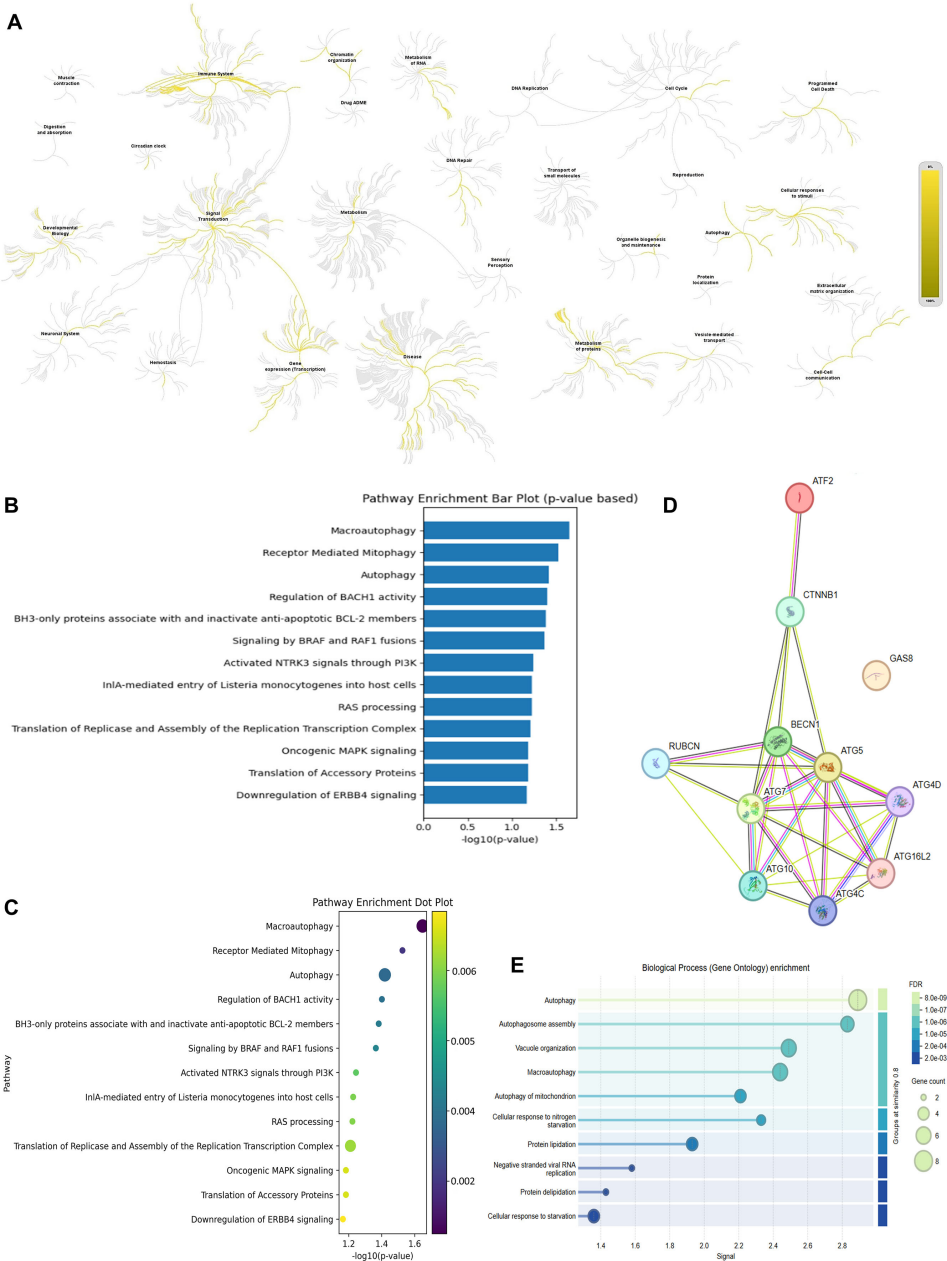
Pathway enrichment analysis of *GAS8-AS1*-related genes in DTCs: REACTOME and STRING. **(A)** This figure presents a genome-wide summary of the REACTOME pathway analysis results. Reactome pathways are organized hierarchically. The center of each circular “burst” represents a top-level parent pathway, such as “autophagy.” Moving outward from the center corresponds to successive lower levels within the pathway hierarchy. The color scale (yellowish gray) indicates the degree of over-representation of each pathway in the input dataset, with light gray denoting pathways that are not significantly over-represented. **(B)** Pathway enrichment bar plot (*p*-value based). The vertical axis lists the pathway names, while the horizontal bars represent the number of interactions. **(C)** Pathway enrichment dot plot. The size of each bubble reflects the number of genes enriched in the corresponding annotation, whereas the color indicates the level of statistical significance. **(D)** Illustration of the regulatory network generated through STRING analysis, in which the network edges visually represent the direction, confidence level, and evidence sources for each regulatory interaction. **(E)** Pathway (Gene Ontology) enrichment using STRING. The vertical list indicates the pathways, while the horizontal line shows the number of interaction signals. The size of each bubble corresponds to the number of genes enriched in the respective annotation, whereas the color reflects the level of statistical significance.

To identify the key signaling involved in the GAS8-AS1-mediated biological regulation process, gene set enrichment for REACTOME and STRING pathway analysis was performed. The REACTOME analysis of DEGs revealed various important vital signaling pathways (**Figure 8A**). Among them, as illustrated in pathway enrichment bar plot (**Figure 8B**) and pathway enrichment dot plot (**Figure 8C**), the pathways of autophagy (macroautophagy, autophagy), translation of replicase and assembly of the replication transcription complex, signaling by BRAF and Raf1 fusion, regulation of BATCH1 activity, BH3-only proteins associate with and inactivate anti-apoptotic BCL-2 members, activated NTRK3 signals through PI3K, RAS processing, oncogenic MAPK signaling, translation of accessary proteins, and downregulation of ERBB4 signaling were significant (ranging, *p* = 2.25 × 10^-2^ – *p* = 6.84 × 10^-2^). Consistently, STRING analysis also revealed that DEGs related to *GAS8-AS1* were mainly enriched in autophagy, macroautophagy, and mitochondrion autophagy. In addition, it also showed autophagosome assembly, vacuole organization, cellular response to nitrogen starvation, protein lipidation, negative-stranded viral RNA replication, protein delipidation, and cellular response to starvation (**Figures 8D, E**). These results indicate that the *GAS8-AS1*-related genes are mainly enriched in autophagy, proliferation, and apoptosis.

## Discussion

We predicted deteriorated RNA secondary structure of *GAS8-AS1* upon its dinucleotide variant, and this was also reflected in our clinical findings (27). Besides, *GAS8-AS1* expression was shown to be critical for *GAS8* tumor suppressive function in hepatic cancer (29), and hence, we speculated that *GAS8-AS1* may be downregulated in DTCs. To ascertain this, we initially analyzed 32 various normal human tissues, including thyroid tissues. We found that the *GAS8-AS1* is highly expressed in normal thyroid tissues, although it is ubiquitously expressed in all normal tissues of other organs. This consecutively prompted us to explore the *GAS8-AS1* expression status in DTCs. The qRT-PCR analysis of twenty-one DTC samples and their twenty-one adjacent normal thyroid tissues from the Saudi population demonstrated a significant downregulation of *GAS8-AS1* in DTCs (paired *t*-test, *p* < 0.0001) compared to that of their paired normal tissues. On the other hand, the paired dot-and-line plot shows that the majority of the paired samples trend in the same direction (normal to DTCs), indicating a marked difference in *GAS8-AS1* expression levels (Wilcoxon signed-rank test, *p* < 0.0001). This consistency confirms that the observed expression pattern is unlikely to be due to random variation. In addition, analysis of RNAseq expression data of 507 DTCs from the TCGA dataset revealed that *GAS8-AS1* was downregulated in 4.9% of DTCs. The discrepancy between our cohort and the TCGA dataset (4.9%) likely arises from methodological and cohort differences. Our study used paired tumor–normal samples analyzed by qRT-PCR, enabling sensitive detection of relative downregulation within the same patient. In contrast, TCGA data are based on RNA-sequencing of largely unpaired samples and rely on predefined, often conservative thresholds, which may underestimate downregulation, particularly for low-abundance lncRNAs such as *GAS8-AS1*. Additionally, differences in cohort composition, tumor heterogeneity, and sample purity may further contribute to this variation. Despite the difference in frequency, both datasets consistently indicate a reduced *GAS8-AS1* expression in DTC. On the other hand, we consistently found that the *GAS8-AS1* is significantly downregulated also in thyroid cancers compared to the normal samples in the Pan-cancer datasets derived from ENCORI. Likewise, expression analysis of various human malignancies also revealed that *GAS8-AS1* is highly downregulated in human cancers except renal and bladder cancers. Moreover, the *GAS8-AS1* downregulation was significantly associated with early-stage disease (*p* = 0.03) and lymph node metastasis (*p* = 0.03) of DTCs. Similarly, *GAS8-AS1* downregulation has also been shown to be associated with early-stage disease in pancreatic cancer (28). Taken together, *GAS8-AS1* is ubiquitously expressed in normal thyroid and deregulated in DTCs and thyroid cancer, which was also reflected in the consortium datasets from ENCORI and TCGA. Besides, its widespread reduction across multiple malignancies suggests that *GAS8-AS1* may function as a common tumor suppressor rather than a cancer type–specific regulator. The extremely low expression observed in ovarian, pancreatic, and thyroid cancers further indicates that loss of *GAS8-AS1* expression could play a critical role in tumorigenesis and may serve as a potential diagnostic and prognostic biomarker in DTCs and other malignancies. In contrast, earlier analyses of *GAS8-AS1* expression in breast and gastric cancers revealed no significant alteration, and the authors suggested that its function may be tissue-specific (28, 46, 47). Consistent with our findings, plasma *GAS8-AS1* levels were significantly reduced in patients with PTC compared to those with nodular goiter. Furthermore, the same group demonstrated that decreased circulating *GAS8-AS1* was independently associated with lymph node metastasis, suggesting its potential utility as a diagnostic and prognostic biomarker in PTC (48).

**Figure 9:**
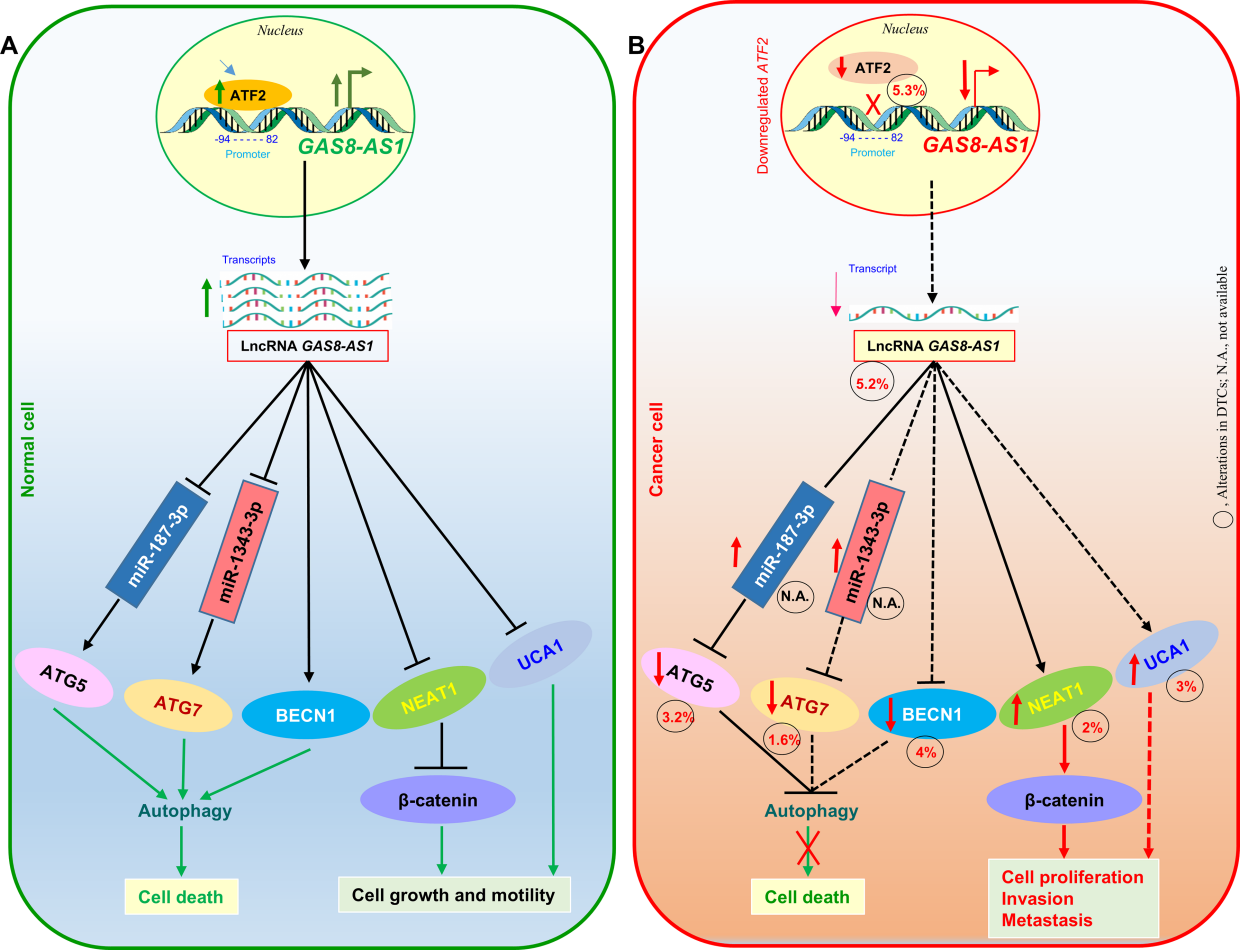
Schematic illustration displays the genetic deregulation of *GAS8-AS1* and its pathway genes in DTCs. **(A)** The illustration shows the signaling of *GAS8-AS1* and its pathway genes in a normal cell. *ATF2* binds in the promoter region of *GAS8-AS1* and triggers its expression, which modulates the expression of downstream candidate genes, including *ATG5*, *ATG7*, *BECN1*, *NEAT1*, and *UCA1* implicated in autophagy, cell growth, and motility. **(B)** Illustration demonstrates the deregulated signaling of *GAS8-AS1* and its pathway genes in a DTC cell. Genetically downregulated *ATF2* mislays promoter binding efficiency, causing loss of expression of *GAS8-AS1*, which downregulates *ATG5*, *ATG7, BECN1*, and upregulates *NEAT1* and *UCA1*, resulting in inhibition of autophagy that promotes cell proliferation, invasion, and metastasis.

ATF2 was shown to activate the expression level of *GAS8-AS1* via ATG5 and ATG7 (41). Besides, BECN1, NEAT1, and UCA1 have been independently reported to be associated with *GAS8-AS1* expression in previous studies (42–44); however, their expression levels have never been analyzed in DTCs. Analysis of the expression level of various genes in the upstream (*ATF2*) and downstream (*ATG5*, *ATG7*, *BECN1*, *NEAT1*, and *UCA1*) of *GAS8-AS1* revealed that the pathway is significantly deregulated (~24%) in DTCs of TCGA. Downregulation of *ATF2*, *ATG7*, and *BECN1* expression was observed in 5.3%, 1.6%, and 4%, respectively, while upregulation of *NEAT1* and *UCA1* was found in 2% and 3%, respectively, in DTCs (**Figure 9**). These results were consistent with all the *ATG5*, *NEAT1*, and *UCA1* genes except *ATG7* and *BECN1* when we analyzed another pan-cancer dataset of thyroid cancer, ENCORI. The *GAS8-AS1* was demonstrated to promote autophagy in ovarian cancer in a *BECN1*-dependent manner (42). Moreover, the *GAS8-AS1* was shown to negatively regulate *NEAT1* to inhibit cell proliferation in glioblastomas (43). The *GAS8-AS1* has also been demonstrated to downregulate *UCA1* to suppress migration and invasion of osteosarcoma cells (44). However, these genes have never been investigated in DTCs/thyroid cancer despite being reported as downstream of *GAS8-AS1* in other malignancies. The *ATG7* and *BECN1* downregulation identified in DTCs of TCGA were not consistent with ENCORI datasets because of the mixed subtypes of thyroid cancer samples. These results suggest that the *GAS8-AS1* pathway genes are significantly deregulated in DTCs and may have a potential role in DTC pathogenesis.

Expression of the transcription factor *ATF2* has been shown to activate *GAS8-AS1* expression to promote autophagy in PTC cells by upregulating the expression of *ATG5* and *ATG7* in thyroid cancer (41). A comprehensive analysis of *DTCs* from *TCGA* datasets revealed that *ATF2* is downregulated in 5% (27/507) cases, indicating that *ATF2* is likely to play a pivotal role beyond autophagy in DTCs and suggested that *ATF2* downregulation tends to alter its downstream interacting molecules. Analysis of the expression level of *ATF2* downstream effectors within the 27 *ATF2*-downregulated DTCs revealed that *GAS8-AS1* (37%), *ATG5* (19%), and *NEAT1* (5%) were remarkably deregulated in an *ATF-2* dependent manner and were significantly correlated with *ATF2* (*ATF2* vs *GAS8-AS1*, *ATG5*, and *NEAT1*). Although *ATG7*, *BECN1*, and *UCA1* were markedly deregulated in DTCs, they were not altered in an ATF2-dependent manner. As >60% of *ATF2*-downregulated samples are deregulated in their pathway members, it is plausible to conclude that *ATF2-*mediated signaling is mainly orchestrated through *GAS8-AS1*, *ATG5*, and *NEAT1*. Though *ATG7*, *BECN1*, and *UCA1* were not deregulated in *ATF2*-downregulated cases, they may have a role in DTCs in an *ATF2*-independent manner (**Figure 9**).

The *GAS8-AS1* dinucleotide alteration was shown to have a significant effect on its expression level (26). In fact, the prevalence of the dinucleotide variant was ~59% in the DTCs of our study. If this variant could affect the expression level of *GAS8-AS1*, a similar prevalence of *GAS8-AS1* downregulation pattern should have been reflected in the DTCs. Invariably, we found a significant *GAS8-AS1* downregulation in DTCs (*p <* 0.0001). In contrast, *GAS8-AS1* expression was found to be downregulated only in 5% of DTCs analyzed from TCGA datasets. These findings suggest that the *GAS8-AS1* dinucleotide variant and downregulated expression pattern may be two independent genetic mechanisms involved in the pathogenesis of DTCs, including thyroid cancer.

The malignant tumorigenesis process mainly turns off most of the genes implicated in anti-proliferative or growth control functions. Interestingly, we found *GAS8-AS1* dinucleotide variant is commonly harbored in our DTC cohort and also found that *GAS8-AS1* is markedly downregulated in DTCs. Transient overexpression of wild-type *GAS8-AS1* was shown to decrease cell viability in PTC cells when compared to the *GAS8-AS1*-bearing dinucleotide variant (26). We, therefore, speculated that *GAS8-AS1* is likely to function as a tumor suppressor by suppressing cell proliferation, migration, and invasion. A siRNA-mediated transient silencing of the *GAS8-AS1* in HEK293T cells robustly promoted cell proliferation and wound healing compared to that of the non-specific siRNA-transfected cells. Further, this was also performed in BCPAP cells, and found that the cells expressing *GAS8-AS1*-specific siRNA dramatically enhanced the proliferation and increased cell invasion compared to that of non-specific siRNA-transfected control cells. Moreover, overexpression of *GAS8-AS1* remarkably suppressed the proliferation of PTC cells, including K1 and BCPAP, supporting its functional role in tumor growth regulation. This was also reflected in qRT-PCR analysis, which showed upregulated expression of *GAS8-AS1* in transfected K1 and BCPAP cells, thereby validating successful transfection. In contrast to siRNA-mediated knockdown, which enhanced proliferation, its overexpression produced the opposite effect, strengthening the evidence for its growth-inhibitory function. These findings demonstrate that *GAS8-AS1* likely acts as a tumor suppressor by negatively regulating cell proliferation pathways. Mechanistically, *GAS8-AS1* appears to exert its effects primarily through the ATG5/NEAT1 axis. Consistent with these findings, overexpression of *GAS8-AS1* has been shown to inhibit cell growth via the miR-135b-5p/CCND2 axis and by negatively regulating the miR-21-3p in PTCs and gastric cancer, respectively (45, 49). Previous studies have shown that the *GAS8-AS1* is regulated through epigenetic activation of *GAS8* in hepatocellular carcinoma. In particular, *GAS8-AS1* was demonstrated to be important for keeping the *GAS8* promoter in an open chromatin condition, which allows recruiting of the MLL1/WDR5 complex to increase RNA polymerase II activity that facilitates the *GAS8* transcription (29). Therefore, *GAS8-AS1* downregulation could probably induce a closed chromatin in the *GAS8* promoter, which may fail to recruit the MLL1/WDR5 complex, causing reduced RNA polymerase activity resulting in loss of *GAS8*-mediated tumor suppression. Moreover, pathway enrichment analysis of *GAS8-AS1*–associated DEGs revealed significant enrichment in pathways related to autophagy, proliferation, and apoptosis, including BRAF, RAF1, NTRK-mediated PI3K signaling, and RAS processing, reflecting the classical PTC–associated cell signaling cascades (6, 7). These findings once again suggest that *GAS8-AS1* likely functions as a tumor suppressor by regulating these canonical signaling pathways. Upon its downregulation, these pathways may become hyperactivated, thereby promoting cellular proliferation, invasion, and inhibiting autophagy and apoptosis.

## Conclusions

*GAS8-AS1* is significantly downregulated, and its reduced expression is strongly associated with early-stage disease and lymph node metastasis in DTCs. Furthermore, *GAS8-AS1* is consistently downregulated across multiple human cancers, including thyroid cancer. Notably, *GAS8-AS1* pathway genes are frequently altered in DTCs (24%) and are enriched in processes that promote proliferation and invasion while suppressing autophagy and apoptosis. The GAS8-AS1, ATG5 and NEAT1 express in an ATF2 dependent manner. Functional assays demonstrated that siRNA-mediated knockdown of *GAS8-AS1* markedly enhances cell proliferation, migration, and invasion in HEK293T and BCPAP cells. On the other hand, a forced overexpression of a mammalian expression vector carrying *GAS8-AS1* significantly reduced the proliferation of PTC cells. This inhibitory effect was consistently observed in both K1 and BCPAP cell lines, supporting its tumor-suppressive role. Gene enrichment analysis revealed that *GAS8-AS1* and its associated pathway genes are closely linked to autophagy, proliferation, invasion, and apoptosis. Collectively, these findings suggest that lncRNA *GAS8-AS1* may function as a tumor suppressor. Moreover, our findings suggest that reduced expression of *GAS8-AS1* is associated with early-stage disease as well as lymph node metastasis in DTCs, and may have potential as a diagnostic and prognostic biomarker. However, these observations warrant further studies to validate the clinical utility of *GAS8-AS1*, including its evaluation through various serum-based biomarker assays.

## Data Availability

The original contributions presented in the study are included in the article/supplementary material. Further inquiries can be directed to the corresponding author.
